# Recognition of mosquito subspecies: the case for *Aedes aegypti* (Diptera: Culicidae)

**DOI:** 10.1093/jme/tjaf067

**Published:** 2025-06-20

**Authors:** Jeffrey R Powell, Andrea Gloria-Soria, John Soghigian

**Affiliations:** Ecology and Evolutionary Biology Department, Yale University, New Haven, CT, USA; Entomology Department, The Connecticut Agricultural Experiment Station, New Haven, CT, USA; Faculty of Veterinary Medicine, University of Calgary, Calgary, AB, Canada

**Keywords:** *Aedes aegypti*, mosquito subspecies

## Abstract

We address the recent proposal by Harbach and Wilkerson to eliminate all Culicidae (mosquito) subspecies names. We defend and promote the use of subspecies for the important vector *Aedes aegypti* (L.): *Aedes aegypti aegypti* and *Ades aegypti formosus.* Harbach and Wilkerson would raise the latter to *Aedes formosus.* We briefly review evidence on this group with relation to various species concepts and find little or no support for this species designation. The 2 forms fit most concepts of subspecies and we advocate continued use of subspecies. Workers on other culicid groups may wish to retain subspecies when accurate, useful, and stable (historical continuity).

Harbach and Wilkerson (2023; hereafter H&W) proposed that subspecies of mosquitoes (Culicidae) should no longer be recognized. This rather radical position, if widely adopted, would bring considerable confusion to mosquito biology and medical entomology in general, particularly for the regional identification of forms of different mosquitoes. Here, we make the case for the retention of subspecies names for one of the most widely studied species of mosquito and one with which we are most familiar: *Aedes aegypti* (L.), from which H&W elevated *Aedes aegypti formosus* to *Aedes formosus*.

*Aedes aegypti* has been a favored model for mosquito research for 3 reasons: (i) it is the major vector for the most important mosquito-borne viruses causing human diseases; (ii) it is very widespread and easy to collect; and (iii) it is the easiest mosquito to rear in the laboratory. The species name “*aegypti*” was first used by Linnaeus in 1762 for a mosquito from Egypt he called *Culex aegypti*, very likely not what we recognize today as *A. aegypti* but rather *Aedes caspius* (Mattingly 1957). In his classic book, Christophers (1960) was able to find 24 Latinized names that up to the year 1900 had been used for this mosquito stating that “...the trouble was that it had so many aliases, almost one for every country and systematist…” Since about 1920, *A. aegypti* has been used almost exclusively.

Mattingly (1957) noted significant differences between *A. aegypti* in sub-Saharan Africa and populations outside Africa. The former have a dark cuticle with limited white or silver scaling on abdominal tergites compared to the lighter-colored form outside Africa that has white scaling on abdominal tergites. Mattingly proposed subspecies names for these different forms, *Ae. aegypti formosus* (here abbreviated Aaf) for the dark African form and *Ae. aegypti aegypti* (Aaa) for the light form outside Africa. Subspecies status seemed appropriate as, at the time, most biologists accepted Mayr’s (1963) definition of subspecies as distinct varieties of a species that are geographically separated. Subsequent studies have confirmed other differences such as the ecology of larval breeding sites and host preferences for blood meals. Extensive genetic studies have confirmed Aaa and Aaf are genetically differentiated (e.g., Gloria-Soria et al. 2016). [A third subspecies proposed by Mattingly, *queenslandensis*, is not genetically distinct from Aaa (Rasic et al. 2016) and is best considered an historical misnomer.]

Importantly, *none of the biological attributes (morphology, ecology, behavior, or genetics) that differ between Aaf and Aaa are absolute.* Morphology is highly variable with no single aspect defining Aaf in Africa and Aaa outside Africa (McClelland 1974). Jupp et al. (1991) were able to rear F_1_ offspring from a single wild-collected female in South Africa that varied in scaling pattern from what would be considered pure Aaf and pure Aaa. While Aaf is generally found in forests or savanna-forest ecotones with larvae in natural pools of water such as tree holes, with the encroachment of human habitats in Africa, what are genetically Aaf (based on genetic data) can be found in artificial containers in villages or cities (e. g., Xia et al. 2021). Mirroring this, Aaa larvae can be found in natural water pools outside Africa (e.g., Chadee et al. 1998). Similarly, while Aaf *tends* to feed from a wide range of mammals and Aaa *tends* to prefer humans for blood meals, this again is not absolute (McBride 2016). Genetic differentiation is the same: all nuclear genetic markers used show marked differentiation between Aaf and Aaa, but with overlap (Fig. 1A–C). [Mitochondrial DNA is not differentiated.] Moreover, phylogenetic analyses consistently demonstrate that Aaa, although monophyletic, is nested within Aaf (Fig. 1D). Most biologists would contend that to designate taxa as different species, there should be some attribute or method of analysis that allows unambiguous assignment to the proper species. This is not the case with Aaa and Aaf.

**Fig. 1.**
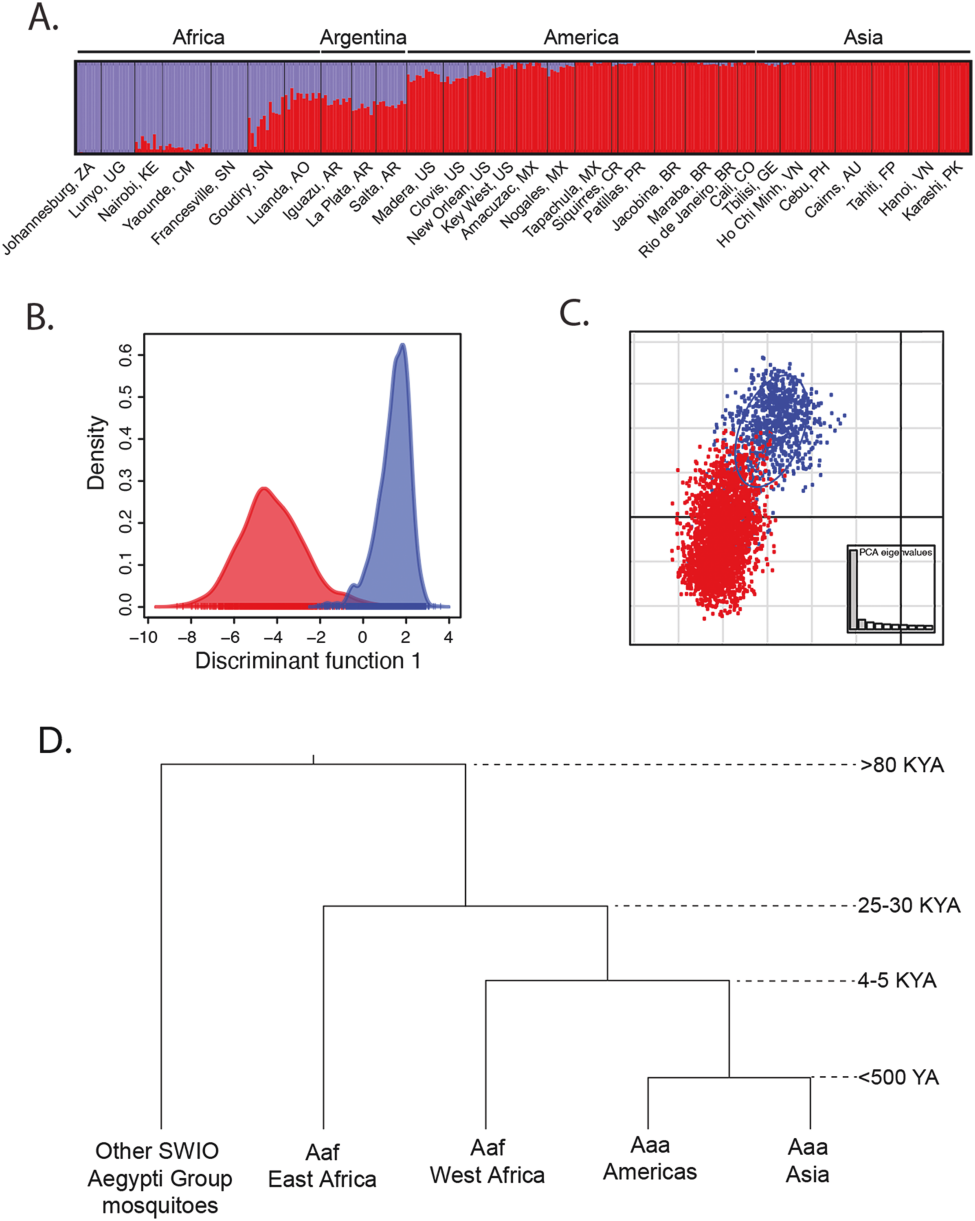
**(A)** Admixture plot for *Aedes aegypti* from Africa (blue) and outside Africa (red) based on 19,000 single nucleotide polymorphisms. Mixed populations in Africa are from Senegal and outside Africa from Argentina. The 2-letter country code following each locality follows the ISO 3166-2 code. (**B**) Discriminant analysis of principal components (DAPC) and (**C**) scatter plot of the first 2 principal components for African (blue) and outside Africa samples (red). **(D)** Phylogeny of *Aedes aegypti* s.l. The Aegypti Group in the southwest Indian Ocean (SWIO) includes at least 2 other species (*pia* and *mascarensis*) as well as *Ae. aegypti*, all basal to continental African *aegypti.* Branch lengths are not to scale. **A** is unpublished data; **B** and **C** are from Gloria-Soria et al. (2016), and **D** is simplified from Soghigian et al. 2020; divergence of human-specialist *Aedes aegypti* and Aaf from Rose et al. 2023.

Beginning with Dobzhansky (1935), a broadly accepted species definition is reproductive isolation: species do not exchange genes. This species concept is widely used in medical entomology. The well-known and extremely important *Anopheles gambiae* s.l. is a prime example. This morphologically uniform complex now has 8 or 9 formally recognized species defined by reproductive isolation. Aaf and Aaa display no signs of reproductive isolation when they come into contact. This is most clearly demonstrated by re-introduction of Aaa into Senegal and coastal Kenya where mixed populations now exist in perfect Hardy-Weinberg proportions (Gloria-Soria et al. 2016). In the lab, mating is random and hybrids (F_1_ and further generations) are viable and fertile.

Thus, by most definitions or concepts of species, Aaa and Aaf would not be considered different species. Why then have H&W proposed the 2 should be called *A. aegypti* and *A. formosus*?

H&W invoke Kevin de Queiroz’s “unified species concept” as being authoritative on the subject of subspecies versus species. De Queiroz (2020) defines subspecies as “…incompletely separated lineages within a more inclusive lineage…” This explicitly places taxonomy in a phylogenetic context where “lineages,” i.e., branches in a phylogenetic tree, are the units to be named. While perhaps controversial to some taxonomists, we have no issues with this, a development only possible as molecular (DNA) data have allowed placing all living beings into reasonably well-supported phylogenies so it is now possible to equate names (formal taxa) to lineages. De Quieroz (2020) continues that “any hypothesized subspecies be supported by the same kinds of evidence that would be required to infer that an entity is a species, as well as evidence that its separation from one or more other species is incomplete.” Aaa and Aaf fit this definition of subspecies: as emphasized above, there is no fixed definitive trait defining Aaa and Aaf in all of their ranges and they remain incompletely separated, yet in much of their ranges they are recognizably distinct. When brought back into contact, they fuse into a single mixed population. Using phylogenies to define a unit to be given a formal species name is also not possible; there is no single “lineage” that includes all Aaf to the exclusion of other taxa (Fig. 1D).

Where de Queiroz and H&W differ from a majority of biologists, ourselves included, is to conclude that this definition of subspecies “…is no longer treated as a taxonomic rank...” It is not completely clear what this means, but these authors seem to be saying subspecies should not be a separate distinct rank from species. But if subspecies are separate units within the more inclusive species unit, there is no logical reason not to consider them a separate rank. Similarly, species are subunits of Genera which are subunits of Families, which are subunits of Orders, etc. The International Code of Zoological Nomenclature (ICZN) recognizes subspecies as subunits within species designated by a trinomial, i.e., 3 latinized names, the species name followed by the subspecies name. De Queiroz (2020) accepts this by stating that “….trinomials are simply a representational device that can (but need not) be used to indicate the nesting of incompletely separated lineages within a more inclusive lineage.” H&W seem to have taken the unified species concept to mean that subspecies are simply species and do not deserve a separate means of designating them, i.e., a binomial is used for both species and subspecies. They would remove trinomials from taxonomy contra both de Queiroz and the ICZN.

As practicing biologist who often use *A. aegypti* in our work, we suggest that names for research organisms should have properties that make them useful in communication. Taxonomy, like language itself, is a human construct that should serve its users. De Queiroz calls subspecies a “representational device” which is what all words are. In this regard, we suggest the names we give to our research subjects should be accurate, useful, and stable. This last property is particularly important for historical continuity so researchers searching the literature for previous work on an organism can easily find relevant publications. When names change, confusion follows. If names are to be changed, these should be agreed upon by workers using the organism to ensure continued accurate communication.

We have made the case for retaining the subspecies designations for *A. aegypti.* We encourage mosquito researchers, journals, and other publications to continue using the subspecific names *Ae. aegypti aegypti* and *Ae. aegypti formosus.* H&W expand their view to all Culicidae. Researchers focusing and publishing on other groups of mosquitoes may well find reasons for retaining subspecies designations to continue accurate and stable communication.

## References

[CIT0001] Chadee D , Ward RA, Novak RJ. 1998. Natural habitats of *Aedes aegypti* in the Caribbean---a review. J. Am. Mosq. Contrl. Assoc. 14:5–11. 9599318

[CIT0002] Christophers R. 1960. Aedes aegypti: the yellow fever mosquito. Cambridge University Press.

[CIT0015] De Queiroz K . 2020. An updated concept of subspecies evolves a dispute about the taxonomy of incompletely separated lineages. Herp. Rev. 51:459–461.

[CIT0003] Dobzhansky T. 1935. A critique of the species concept in biology. Phil. Sci. 2:344–355. https://doi.org/10.1086/286379

[CIT0004] Gloria-Soria A , Ayala D, Bheecarry A, et al 2016. Global genetic diversity of *Aedes aegypti*. Mol. Ecol. 25:5377–5395. https://doi.org/10.1111/mec.13866 27671732 PMC5123671

[CIT0005] Harbach RE , Wilkerson RC. 2023. The insupportable validity of mosquito subspecies (Diiptera: Culicidae) and their exclusion from culicid classification. Zootaxa. 5303:1–184. https://doi.org/10.11646/zootaxa.5303.1.1 37518540

[CIT0006] Jupp PG , Kemp A, Frangos C. 1991. The potential for dengue in South Africa: morphology and taxonomic status of *Aedes aegypti* populations. Mosq. Syst. 23:182–190.

[CIT0007] Mattingly PF. 1957. Genetical aspects of the *Aedes aegypti* problem. I. Taxonomy and bionomics. Ann. Trop. Med. Parasitol. 51:392–408. 13498658

[CIT0008] Mayr E. 1963. Animal species and evolution. Harvard University Press.

[CIT0009] McBride CS. 2016. Genes and odors underlying the recent evolution of mosquito preference for humans. Curr. Biol. 26:R41–R46. https://doi.org/10.1016/j.cub.2015.11.032 26766234 PMC4714039

[CIT0010] McClelland GAH. 1974. A worldwide survey of variation in scale pattern of the abdominal tergum of *Aedes aegypti* (L.) (Diptera: Culicidae). Trans. R. Entomol. Soc. Lond. 126:239–259. https://doi.org/10.1111/j.1365-2311.1974.tb00853.x

[CIT0011] Rasic G , Filipovic I, Callahan AG, et al 2016. The *queenslandensis* and the type form of the dengue mosquito (*Aedes aegypti*) are genomically indistinguishable. PLoS Negl.Trop. Dis. 10:e0005096. https://doi.org/10.1371/journal.pntd.0005096 27806047 PMC5091912

[CIT0012] Rose NH , Badolo A, Sylla M, et al 2023. Dating the origin and spread of specialization on human hosts in *Aedes aegypti* mosquitoes. eLife. 12:e83524. https://doi.org/10.7554/eLife.83524 36897062 PMC10038657

[CIT0013] Soghigian J , Gloria-Soria A, Robert V, et al 2020. Genetic evidence for the origin of *Aedes aegypti,* the yellow fever mosquito, in the southwestern Indian Ocean. Mol. Ecol. 29:3593–3606. https://doi.org/10.1111/mec.15590 33463828 PMC7589284

[CIT0014] Xia S , Dweck HKM, Lutomiah J, et al 2021. Larval breeding sites of the mosquito *Aedes aegypti* in forest and domestic habitats in Africa and the potential association with oviposition evolution. Ecol. Evol. 11:16327–16343. https://doi.org/10.1002/ece3.8332 34824830 PMC8601902

